# External Beam Radiotherapy in Advanced Pheochromocytoma and Paraganglioma: An Observation of a Rare Abscopal Effect

**DOI:** 10.1210/jcemcr/luad111

**Published:** 2023-09-21

**Authors:** Aiko Terakawa, Akiyo Tanabe, Hidetsugu Nakayama, Ryogo Minamimoto

**Affiliations:** Department of Diabetes, Endocrinology and Metabolism, National Center for Global Health and Medicine, Tokyo, 162-8655, Japan; Department of Diabetes, Endocrinology and Metabolism, National Center for Global Health and Medicine, Tokyo, 162-8655, Japan; Department of Radiation Oncology, National Center for Global Health and Medicine, Tokyo, 162-8655, Japan; Division of Nuclear Medicine, Department of Radiology, National Center for Global Health and Medicine, Tokyo, 162-8655, Japan

**Keywords:** external beam radiotherapy, metastatic pheochromocytoma and paraganglioma, abscopal effect, catecholamine

## Abstract

Metastatic pheochromocytoma and paraganglioma are incurable, and effective treatment of systemic symptoms resulting from catecholamine excess and local symptoms from tumor growth are crucial to prolong survival and improve quality of life. We report the first case of metastatic pheochromocytoma wherein external beam radiotherapy (EBRT) demonstrated efficacy in both target and nontarget lesions, demonstrating the “abscopal effect.” EBRT reduced tumor volume and catecholamine secretion and improved catecholamine excess-related complications, including glycemic control. EBRT is an effective treatment option for metastatic pheochromocytoma and paraganglioma because of its minimal invasiveness, safety, and potential for the rare abscopal effect.

## Introduction

Pheochromocytoma (PCC) and paraganglioma (PPGL) are rare catecholamine-secreting neuroendocrine tumors that arise from chromaffin cells. PCC and paraganglioma are derived from the adrenal medulla and extra-adrenal paraganglia, respectively.

PPGLs have malignant potential, and metastatic disease is found in 10% to 17% of PPGL cases. Malignancy is confirmed when metastases are present in nonchromaffin tissues, such as the bone, lymph nodes, lungs, and liver. The prognosis of metastatic PPGL is poor; the 5-year and 20-year survival rates in nonmetastatic PPGLs are 96.8% and 93.7% [1], respectively, whereas, in metastatic PPGLs, these survival rates are 84.7% and 57.3% [1], respectively. Therefore, treating systemic symptoms resulting from catecholamine excess and local symptoms from tumor growth in patients with metastatic PPGL is crucial to prolong survival and improve quality of life. Curative treatment options for metastatic PPGL are currently unavailable; therefore, patients are treated with multidisciplinary therapies such as debulking surgery, chemotherapy (including cyclophosphamide, dacarbazine, and vincristine [CVD] regimen, tyrosine kinase inhibitors, and immunotherapeutic agents), and the radionuclides ^131^I-metaiodobenzylguanidine or ^177^Lu-DOTATATE. For local tumor control, external beam radiotherapy (EBRT), ablation therapy, and chemoembolization have been used.

EBRT is frequently used for local control and relief of metastatic PPGL-related bone pain and spinal cord compression. Local control has been achieved in 76% to 87% of patients, local symptom improvement in 81% to 94% of patients undergoing EBRT without severe toxicity [2, 3], and nonirradiated tumor progression in 75% of patients [3].

However, limited reports have evaluated the isolated efficacy of EBRT because its effects are not immediate and PPGLs are treated with multiple therapeutic modalities. Here, we report a patient with metastatic PCC in which EBRT demonstrated efficacy in both target and nontarget lesions, demonstrating the “abscopal effect,” with significantly reduced tumor size and decreased 24-hour urine normetanephrine (u-NMN) excretion.

## Case Presentation

### Case 1

A 46-year-old Japanese man had an abdominal computed tomography (CT) scan that incidentally discovered a left adrenal tumor measuring 13 × 10 × 5 cm. His 24-hour u-NMN and metanephrine levels were 213.4 µmol/day (39.1 mg/day) (reference range, <1.6 µmol/day; <0.3 mg/day) and 1.5 µmol/day (0.3 mg/day) (reference range, <1.0 µmol/day; < 0.2 mg/day), respectively. The measurement of plasma free fractionated metanephrines was not available in Japan. Whole-body ^131^I-metaiodobenzylguanidine scintigraphy showed uptake was limited to the tumor site. He had diabetes mellitus with a glycated hemoglobin (HbA1c) level of 8.9%.

## Treatment

Alpha-adrenergic blockade was initiated as preoperative treatment, although despite the markedly elevated 24-hour u-NMN excretion, he did not have persistent or paroxysmal hypertension. After left adrenalectomy, his 24-hour u-NMN excretion normalized and the α-adrenergic blockade was discontinued. The HbA1c level improved to 5.0% without the aid of diabetes mellitus medications. We evaluated creatinine-corrected spot urine metanephrines, HbA1c level, and imaging findings in outpatient follow-up.

**Figure 1.**
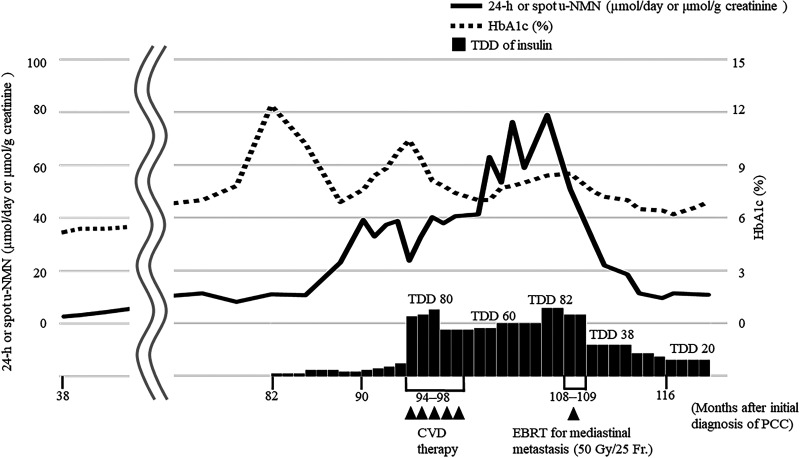
Clinical course after primary tumor resection in case 1. Small lung metastases appeared 38 months after the initial diagnosis of pheochromocytoma. Spot u-NMN levels gradually increased. Because HbA1c levels suddenly deteriorated to 12.4% at 82 months, insulin therapy was initiated. Subsequently, mediastinal lymph node metastases appeared. Spot u-NMN levels increased to 38.2 µmol/g creatinine (7 mg/g creatinine), and insulin requirements increased to 80 units/day. Five courses of CVD chemotherapy were ineffective. EBRT for mediastinal metastases was administered at 108 months. On the 15th day following EBRT, glycemic control improved, and the daily insulin doses were reduced to 38 units per day. Six months after EBRT, u-NMN and HbA1c levels were maintained at 10.9 µmol/g creatinine (2 mg/g creatinine) and 6.5% to 6.9% with 20 units of insulin per day, respectively. CVD, cyclophosphamide, dacarbazine, and vincristine; EBRT, external beam radiotherapy; HbA1c, glycated hemoglobin; TDD, total daily insulin dose; u-NMN, urine normetanephrine.

Thirty-eight months after the initial PCC diagnosis, multiple nodular lung lesions appeared, which were pathologically confirmed as PCC metastases (Fig. 1). The metastatic lesions grew slowly over the subsequent 44 months. At 82 months after PCC resection, the disease progressed rapidly, with spot u-NMN and HbA1c levels increasing to 10.9 µmol/g creatinine (2.0 mg/g creatinine) (reference range, < 1.6 µmol/g creatinine; < 0.3 mg/g creatinine) and 12.4%, respectively. Alpha-adrenergic blockade was initiated, and multiple daily insulin injections were required to achieve glycemic control. Additionally, multiple new metastases were identified in the mediastinal lymph nodes. Although the patient underwent 5 cycles of chemotherapy with the CVD regimen, increased 24-hour u-NMN excretion (40.4 µmol/day; 7.4 mg/day) and enlarging metastatic lesions in the lung and mediastinal lymph nodes on ^18^F-fluorodeoxyglucose-positron emission tomography (FDG-PET)/CT indicated progressive disease. The patient declined to continue chemotherapy. Because radionuclide treatment was unavailable in Japan at that time, no other systemic treatment options were available for the patient. The metastatic disease was limited to the lungs and mediastinal lymph nodes.

**Figure 2.**
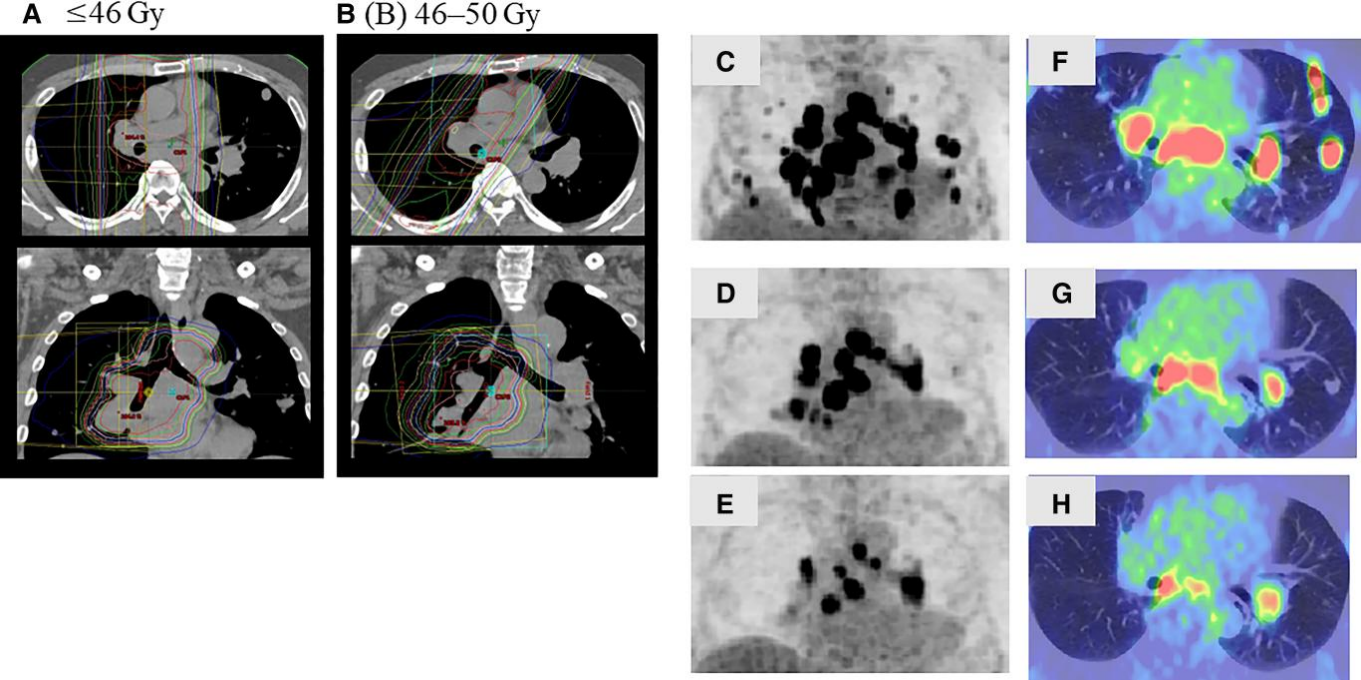
Dosimetry of 3D conformal radiotherapy (A, B) and ^18^F-FDG-PET (C-E) and ^18^F-FDG-PET/CT (F-H) findings 2 months before EBRT (C, F), 6 months (D, G), and 10 months (E, H) after EBRT. The lesions were treated using a 3D conformal radiotherapy total dose of 50 Gy administered in 25 fractions (dosimetry of ≤46 Gy and 46-50 Gy is shown in A and B, respectively). ^18^F-FDG-PET/CT showed shrinkage of the right mediastinal lymph nodes within the irradiated area with a decreasing SUV. Additionally, the left hilar lymph nodes and multiple lung metastases outside the irradiated area also decreased in size or disappeared. EBRT, external beam radiation therapy; 3D, 3-dimensional; FDG-PET, ^18^F-fluorodeoxyglucose-positron emission tomography; CT, computed tomography; SUV, standardized uptake value.

At 108 months postoperatively, his spot u-NMN and HbA1c levels were 50.8 µmol/g creatinine (9.3 mg/g creatinine) and 8.4%, respectively. For his diabetes, he required treatment with 82 units of insulin per day. The patient underwent EBRT to limit the impact of the enlarging mediastinal lymph nodes on the surrounding mediastinal tissues. The extent of the external radiation exposure is shown in Fig. 2. The lesions were treated with EBRT using a 3-dimensional conformal technique with a total dose of 50 Gy in 25 fractions; adverse events were absent.

## Outcome and Follow-up

**Figure 3.**
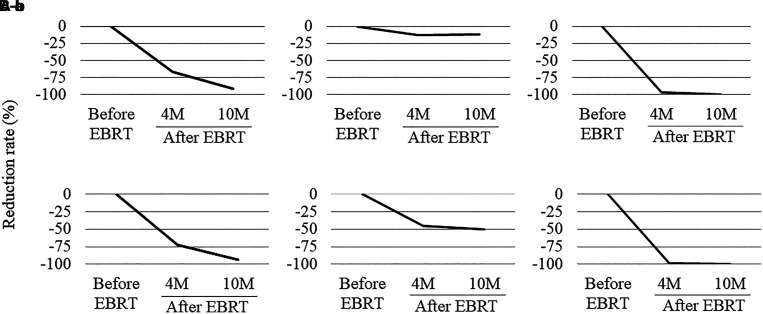
The reduction rate of (A) MTV and (B) TLG. The changes in MTV before EBRT and 4 and 10 months after EBRT were 87.84, 29.79, and 7.99 mL (A-a), 13.06, 11.42, and 11.48 mL (A-b), and 18.66, 0.72, and 0.15 mL (A-c), respectively. The changes in TLG before EBRT and 4 and 10 months after EBRT were 641.03, 177.89, and 39.02 (B-a), 89.92, 49.2, and 44.59 (B-b), and 84.59, 1.29, and 0.15 (B-c), respectively. EBRT, external beam radiation therapy; MTV, metabolic tumor volume; TLG, total lesion glycolysis.

^18^F-FDG-PET/CT scans completed before and after EBRT demonstrated tumor volume reduction (Fig. 2). We evaluated the metabolic tumor volume, an index that reflects the size and extent of tissues with high glucose metabolism and tumor lesion glycolysis, which is calculated by multiplying metabolic tumor volume with the average value of the standardized uptake value as an FDG accumulation index [4]. The sequential reduction rates of these indices are shown in Fig. 3. Interestingly, this effect was observed in both target (ie, metastatic right-sided mediastinal lymph nodes) and nontarget lesions (ie, metastatic left hilar lymph nodes and lung metastases). The spot u-NMN levels gradually declined, and the HbA1c levels improved after EBRT. Seven months after EBRT, the daily insulin requirement dropped to 20 units per day. In addition, the spot u-NMN and HbA1c levels were 10.9 µmol/g creatinine (2.0 mg/g creatinine) and 6.5%, respectively. There was no tumor progression for more than a year after EBRT.

### Cumulative experiences of EBRT in PPGL

**Figure 4.**
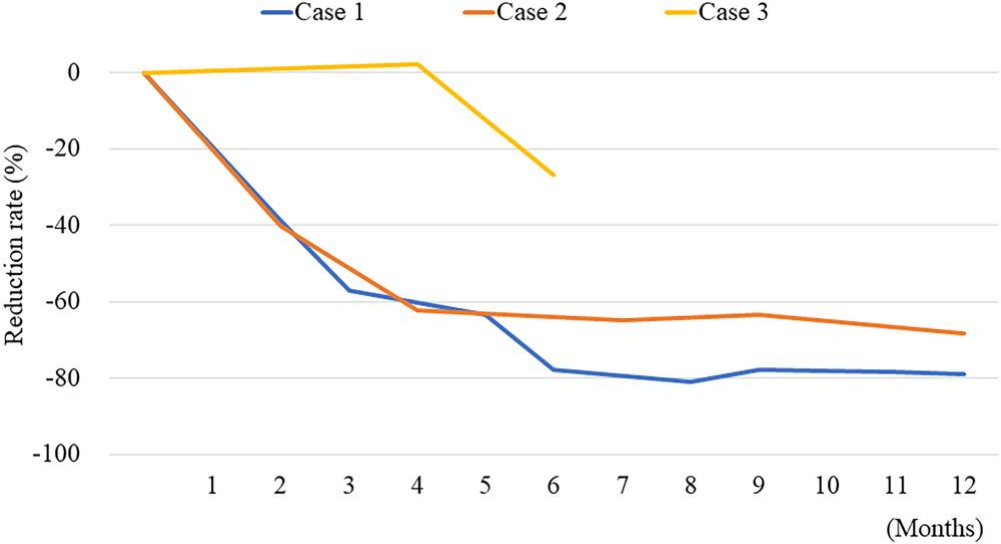
Reduction rate of u-NMN levels after EBRT in 3 cases. u-NMN levels sequentially decreased after EBRT in all cases. EBRT, external beam radiotherapy; u-NMN, urine normetanephrine.

During the evaluation and treatment (2016-2021) of our patient, there were 8 patients with metastatic PPGL who received EBRT using the 3-dimensional conformal technique in our hospital, and 3 of them (including our patient presented here) did not receive additional treatments for more than 2.5 months before and 6 months after EBRT (Table 1). The 3 patients did not have family history of PPGL, and genetic testing was not performed because of cost and lack of insurance coverage. The 24-hour or spot u-NMN levels gradually increased before EBRT in all cases during treatment with α-adrenergic blockade. EBRT methods are shown in Table 2. The average EBRT dose and biological effective dose (α/β = 10) (BED_10_) were 42 and 42.8, respectively. We defined an increase in tumor volume as an increase of ≥50% in the known tumor's diameter or as the appearance of new tumors and a decrease as a reduction of ≥50% in the known tumor's diameter on contrast-enhanced CT. An increase in HbA1c of ≥0.5% was defined as deterioration and a decrease of ≥0.5% was defined as an improvement. Tumor growth did not occur in any targeted lesion during the observation period of 6 to 26 months. However, reduction in size of nontarget lesions was only observed in the 1 patient reported here (Table 2). The 24-hour u-NMN levels decreased in all cases after EBRT (Table 3 and Fig. 4).

**Table 1.** Clinical features of 3 patients with PPGL who were treated with EBRT following no additional treatment for >6 months

	Age/sex	PCC or PGL	Disease duration	Biochemical phenotype	Treatments before EBRT	Sites of metastases	Duration since last treatment
Case 1	55/M	PCC	8 y	Norepinephrine	Primary tumor resection CVD 4 cycles	Mediastinal lymph nodes Lung	10 mo (CVD)
Case 2	72/M	PGL	5 y	Norepinephrine	Primary tumor resection CVD 19 cycles	Thoracic spine	2 y (CVD)
Case 3	52/F	PCC	32 y	Norepinephrine	Primary tumor resection Right lung metastases resection Right renal metastases resection CVD 3 cycles EBRT for left mandible	Mediastinal lymph nodes Left mandible Lumber spine Right retroperitoneum	8 mo (EBRT) 2.5 mo (CVD)

Abbreviations: CVD, chemotherapy with cyclophosphamide, dacarbazine, and vincristine; EBRT, external beam radiotherapy; F, female; M, male; PCC, pheochromocytoma; PGL, paraganglioma; PPGL, pheochromocytoma and paraganglioma.

**Table 2.** Methods of EBRT and changes in tumor volume in target and nontarget lesions

	Target sites of EBRT	Gy/Fr	BED_10_ (Gy)	Volume change (observation periods after EBRT)	
				Target lesions	Nontarget lesions
Case 1	Right mediastinal lymph nodes	50 Gy/25 Fr	50.0	Decrease (11 mo)	Lung metastases: decrease (11 mo) Left hilar lymph nodes: decrease (11 mo)
Case 2	Thoracic spine	46 Gy/23 Fr	46.0	No change (26 mo)	None
Case 3	Lumber spine right and retroperitoneum	30 Gy/10 Fr	32.5	No change (7 mo in lumber spine and 6 mo in right retroperitoneum)	Right hilar lymph nodes: increase (6 mo) Left mandible: no change (6 mo)

Abbreviations: BED_10_, biological effective dose (α/β=10); EBRT, external beam radiotherapy.

**Table 3.** Changes in urine normetanephrine and glycemic control before and after EBRT

	u-NMN		Glycemic control			
	Before EBRT	After EBRT (time of evaluation after EBRT)	Before EBRT		After EBRT (time of evaluation after EBRT)	
			HbA1c	Treatments	HbA1c	Treatments
Case 1	50.8 µmol/gCre (9.3 mg/gCre)	10.9 µmol/gCre (2.0 mg/gCre) (12 mo)	8.5%	DPP4i + insulin 82 units	6.9%	DPP4i + insulin 20 units (12 mo)
Case 2	8.7 µmol/gCre [New line](1.6 mg/gCre)	3.3 µmol/gCre (0.6 mg/gCre) (12 mo)	6.8%	No medications	6.1%	No change (12 mo)
Case 3	31.1 µmol/gCre (5.7 mg/gCre)	22.9 µmol/gCre (4.2 mg/gCre) (6 mo)	6.2%	DPP4i + TZD + SU	6.3%	No change (6 mo)

Abbreviations: Cre, creatinine; DPP4i, dipeptidyl peptidase-4 inhibitor; EBRT, external beam radiotherapy; HbA1c, glycated hemoglobin; NA, not assessed; SU, sulfonylureas; TZD, thiazolidine; u-NMN, spot urine normetanephrine.

## Discussion

This is the first case report of the abscopal effect on metastatic PPGL. In 1953, Mole et al [5] first described the abscopal effect as “action at a distance from the irradiated volume.” Although the mechanism remains unknown, the abscopal effect is likely mediated by antitumor immune response activation through radiotherapy. The abscopal effect has been documented in 46 cases reported between 1969 and 2014 [6]. Recently, the abscopal effect has attracted attention with the advent of immune checkpoint inhibitors (ICIs), resulting in an increase in the number of studies on the abscopal effect in patients treated with combination therapy of EBRT and ICIs, particularly in melanomas and non–small cell lung carcinomas. Systematic reviews have reported that the abscopal effect is observed in 1% to 45% of patients receiving combination therapy of EBRT and ICIs [7]. However, predictors of the duration of the abscopal effect and survival remain to be determined [8], and no reports exist on the abscopal effect in patients with metastatic PPGL. It has been reported that germline mutations in genes that predispose to PPGL may affect response to some treatments and prognosis. The relationships between germline pathogenic variants in PPGL genes and tumor response to EBRT or abscopal effect are unclear. Genetic testing is still not common in Japan because of cost and lack of insurance coverage; therefore, our patients described here did not have germline genetic testing.

EBRT decreases catecholamine secretion from target tumors, even in cases without significant tumor volume reduction. Previous reports revealed biochemical responses in 50% to 100% of cases in small groups of 4 to 6 patients [2, 3]. Breen et al reported that patients receiving BED_10_ ≥ 53 had higher local control rates than those receiving BED_10_ ≤ 53, and no acute grade ≥3 treatment-related adverse events were observed in patients with BED_10_ between 9 and 132 [2]. In the patient reported here and in the other patients treated at our hospital during the same period, a biochemical response was observed in all patients, with an average BED_10_ of 42.8 (range, 32.5-50) and without any acute adverse events. EBRT-induced reduction in catecholamine secretion improved the catecholamine excess-associated complications. Additionally, glycemic control improved in concert with reduction in 24-hour u-NMN excretion after EBRT in 2 of the 3 patients in whom HbA1c levels were monitored. Although an early response to EBRT is rare, glycemic monitoring is necessary for patients treated with hypoglycemic agents.

A case of metastatic paraganglioma with exacerbated hypertension after EBRT has been reported [9]; however, several reports suggested no EBRT-related acute adverse events [2, 3]. In contrast, other local treatments, such as ablative therapy and chemoembolization, can cause severe complications, including hypertensive crises [10]. Therefore, EBRT is a safe local treatment option for patients with metastatic PPGL. Furthermore, EBRT may be a preferred option for both slowly progressing and limited metastatic PPGLs and for frail patients with advanced tumors who cannot undergo systemic therapy.

## Learning Points

- External beam radiotherapy (EBRT) reduces tumor volume and catecholamine secretion and improves catecholamine excess-related complications, including glycemic control, in patients with metastatic pheochromocytoma and paraganglioma (PPGL).
- EBRT is an effective treatment option for patients with metastatic PPGL because of its minimal invasiveness, safety, and potential for the rare abscopal effect.

## Data Availability

Original data generated and analyzed during this study are included in this published article.

## Abbreviations

BED_10_: biological effective dose (α/β = 10)
CT: computed tomography
CVD: cyclophosphamide dacarbazine, and vincristine
EBRT: external beam radiotherapy
HbA1c: glycated hemoglobin
ICI: immune checkpoint inhibitor
PCC: pheochromocytoma
PPGL: pheochromocytoma and paraganglioma
u-NMN: urine normetanephrine
